# An appeal to our government for nationwide policies in the prevention of cardiovascular disease

**DOI:** 10.1007/s12471-021-01628-w

**Published:** 2021-10-04

**Authors:** T. J. van Trier, N. Mohammadnia, M. Snaterse, R. J. G. Peters, H. T. Jørstad, W. A. Bax, J. D. Mackenbach

**Affiliations:** 1 Department of Cardiology, Amsterdam University Medical Centre, University of Amsterdam, Amsterdam, The Netherlands; 2 Department of Internal Medicine, Northwest Clinics, Alkmaar, The Netherlands; 3 Vascular Research Alkmaar, Alkmaar, The Netherlands; 4 Department of Cardiology, Northwest Clinics, Alkmaar, The Netherlands; 5 Centre of Expertise Urban Vitality, Faculty of Health, Amsterdam University of Applied Sciences, Amsterdam, The Netherlands; 6 Department of Epidemiology and Data Science, Amsterdam Public Health Research Institute, Amsterdam UMC, location VUmc, Amsterdam, The Netherlands

**Keywords:** Lifestyle, Health behaviour, Public health, Preventive medicine, Government, Smoke-free policy

## Abstract

The high prevalence and burden of cardiovascular diseases (CVD) is largely attributable to unhealthy lifestyle factors such as smoking, alcohol consumption, physical inactivity and unhealthy food habits. Prevention of CVD, through the promotion of healthy lifestyles, appears to be a Sisyphean task for healthcare professionals, as the root causes of an unhealthy lifestyle lie largely outside their scope. Since most lifestyle choices are habitual and a response to environmental cues, rather than rational and deliberate choices, nationwide policies targeting the context in which lifestyle behaviours occur may be highly effective in the prevention of CVD. In this point-of-view article, we emphasise the need for government policies beyond those mentioned in the National Prevention Agreement in the Netherlands to effectively reduce the CVD risk, and we address the commonly raised concerns regarding ‘paternalism’.

**Supplementary Information:**

The online version of this article (10.1007/s12471-021-01628-w) contains supplementary material, which is available to authorized users.

## Introduction

**Fig. 1:**
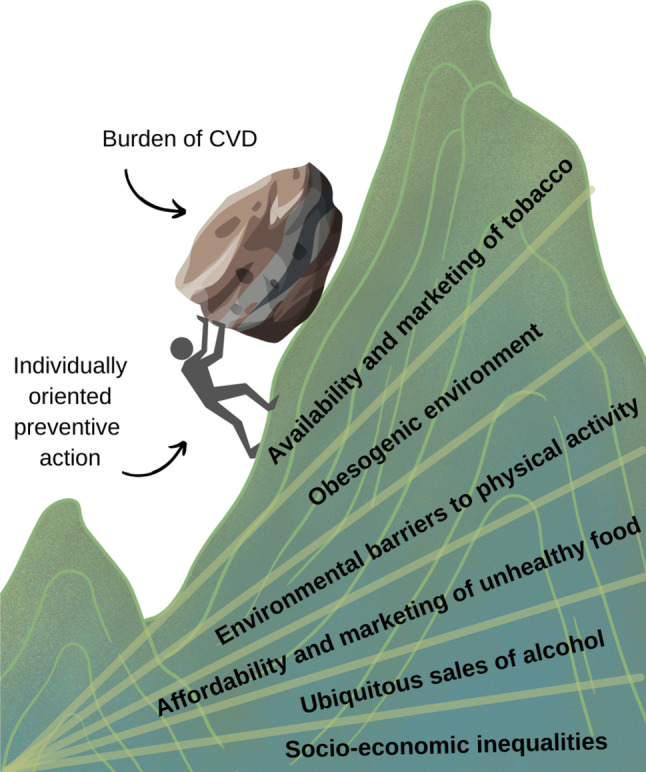
The laborious and unrewarding task of promoting healthy lifestyle in an atherogenic environment can be symbolised as a Sisyphean task. Adapted from ‘The health gradient’ [36]

Despite improvements in the detection and treatment of cardiovascular disease (CVD), CVD remains the leading contributor to the burden of disease in the Netherlands and worldwide. Dietary risks, tobacco use, alcohol consumption and low physical activity are ranked in the top 12 modifiable risk factors for CVD, which have barely changed since 1990 [1]. The high prevalence, disease burden and socio-economic inequalities in CVD, and the prominent role of an unhealthy lifestyle [1, 2], call for an increased focus on the prevention of CVD. Yet, in an environment that promotes the consumption of alcohol, cigarette smoking, sedentary behaviours and overeating, the promotion of a healthy lifestyle by individual healthcare professionals is a Sisyphean task (Fig. 1).

Healthcare professionals are confronted with the results of an unhealthy lifestyle on a daily basis, but their impact on lifestyle modification is limited and temporary. Along with the opportunity to influence individual lifestyles, physicians are typically called upon as ‘firefighters’ when prevention has failed, for example when stenting a coronary artery occlusion causing acute myocardial infarction. In addition to addressing the end stage of lifestyle-related disease, such as acute coronary syndrome, we now call upon the government to address the root causes of lifestyle behaviour through policies that promote and sustain a population-wide healthier lifestyle [3, 4]. In other words, rather than strengthening the muscles of Sisyphus to push the rock up the hill, we should metaphorically flatten the hill.

However, population-level preventive policies are beyond the influence of healthcare professionals. Primordial prevention (which involves preventing the development of risk factors for disease), primary prevention (which requires modifying existing risk factors to prevent the development of disease) and even secondary prevention (to prevent progression or recurrence of disease) all take place largely outside the healthcare sector. Ideally, collaborative action should be taken by stakeholders, including industry. However, due to misalignment of interests (profit vs public good), the involvement of commodity industries in policy making often results in paradoxical policy inertia [5]. Upstream, population-wide interventions therefore mainly depend on government action [6]. This corresponds to the notion that the government is obliged by Article 22 of Dutch constitutional law to promote public health for its citizens. A well-known example of such government-level, nationwide intervention can be found in North Karelia, where an 84% reduction in coronary mortality was achieved through long-term, population-based intervention strategies such as reducing the salt content of food and a ban on tobacco advertising [7]. In this point-of-view article, we describe examples of government policies that may effectively contribute to the prevention of CVD, ranging from ‘softer’ policies (steering measures) to ‘harder’ policies (taxes and bans). These population-based policies may affect the whole spectrum of individuals at risk for CVD, which is why we refrain from making a distinction between different stages of prevention from this point onwards. We conclude by addressing the commonly raised concern regarding ‘paternalism’ with respect to government intervention in public health.

## ‘Soft’ policies

Health education may engage individuals in healthy behaviour, just as dentists succeeded in promoting tooth brushing. Mass media campaigns have been used widely to reduce alcohol and tobacco consumption and to promote healthy food habits and physical activity. A Cochrane review concluded that mass media campaigns can effectively contribute to reducing smoking rates in adults, although evidence was obtained from studies of variable quality [8]. A US food policy model suggested that a 1-year mass media campaign in 2015 targeting fruit and vegetable consumption would prevent 18,600 CVD deaths by 2030 (95% confidence interval (CI): 17,600–19,500) [9]. The main advantage of mass media campaigns is the ability to reach a large population repeatedly over time at low cost. However, poor design, limited exposure or addressing the wrong behaviour may limit their impact [10]. Also, health education and mass media campaigns require individual action, which limits their success in those in lower socio-economic positions. There is evidence that suggests that ‘social marketing’ or ‘health branding’ approaches—i.e. using marketing principles to promote healthy choices—may be effective in supporting healthy eating, although robust evaluations are lacking [11]. In this context, the European Tobacco Products Directive (2014/40/EU) requires health warnings on tobacco products. The use of nutritional or warning labels, mandatory or not, may contribute to healthier food and beverage intake and lower alcohol and tobacco consumption, although this depends on the type and positioning of the label. For example, 3.4% of all diet-related non-communicable disease mortality is estimated to be avoidable when the ‘Nutri-Score’ is used as a front-of-pack nutrition label, while this would be only 1.6% for the ‘Multiple Traffic Light’ label [12].

Choice architecture [13] or nudging [14] interventions are gaining popularity, since their liberty-preserving character is well aligned with the current neoliberal political climate in the Netherlands. Nudging interventions such as stair prompts [15] (e.g. through directional footprints on the floor) and repositioning healthy foods or making them more salient [16] may have modest effects on cardiovascular risk factors, yet clinical outcome data are lacking. Another ‘soft’ opportunity lies in the use of individual financial incentives to promote a healthy lifestyle. For instance, smoking cessation programmes based on obtaining rewards are considered acceptable, although programmes based on the possibility of losing a deposit may be more efficacious [17]. Yet, the effects of such financial incentives on promoting physical activity appear to be limited [18].

## ‘Hard’ policies

The ‘soft’ policies mentioned above compete with advanced marketing and sales of unhealthy commodity industries. For instance, considering the deadly and addictive design of cigarettes, stronger policies such as taxes, advertising and sales bans are necessary to counteract the commercial push. Comprehensive advertising bans in various settings (schools, hospitals, restaurants) have been associated with significant reductions in smoking rates [19]. As part of the National Prevention Agreement in the Netherlands, the visible display of tobacco products in stores has not been allowed since 2020. Still, the Dutch Institute for Public Health and the Environment concluded that additional measures on top of the National Prevention Agreement are needed to protect children and pregnant women from the harmful effects of smoking [20]. Institutional smoking bans have also led to reduced smoking rates in hospitals (odds ratio (OR) 0.76; 95% CI: 0.69–0.81) and universities (OR 0.72; 95% CI: 0.64–0.80) [21]. In prisons, there was a 9% reduction in smoking-related mortality (incidence ratio 0.91; 95% CI: 0.88–0.95) [22]. The smoke-free legislation in the United Kingdom—prohibiting smoking in all enclosed public areas and workplaces—has contributed to an estimated 1200 fewer hospital admissions for myocardial infarctions in the 1st year [23].

In 1776, Adam Smith noted that ‘sugar, rum and tobacco are commodities which are nowhere necessaries of life …which are …objects of almost universal consumption, and which are therefore extremely proper subjects of taxation’ [24]. Currently, substantial and regular increases in the price of tobacco products are considered the most effective policy measure to reduce smoking, including in people in low socio-economic positions [25, 26]. Similarly, higher alcoholic beverage tax is associated with lower alcohol consumption, as regards both frequency and intensity [26], and sugar-sweetened beverage taxation reduces sugar-sweetened beverage sales [27]. The same US food policy model that estimated the impact of a mass media campaign suggested that a 10% reduction in the price of fruit and vegetables would prevent 153,300 CVD deaths (95% CI: 146,400–159,200) [9]. While neither a subsidy on fruit and vegetables nor a sugar-sweetened beverage tax is currently included in the National Prevention Agreement in the Netherlands [20], the National Institute for Public Health and the Environment listed them as a top priority for additional measures in the prevention of obesity and associated chronic diseases [28]. A simulation study in the United Kingdom estimated that prohibiting television advertising of unhealthy foods and beverages during daytime would result in 40,000 fewer children with obesity, ultimately possibly averting the loss of 240,000 disability-adjusted life years [29]. Finally, more intense alcohol licensing policies can contribute to a 5% reduction in alcohol-related admission rates [30].

## Government interference vs freedom of choice

The extent to which governments interfere may depend on the political climate. The present neoliberal climate in the Netherlands relies on the market as a social ordering mechanism, assuming that, within a market, people are free and capable to make their own choices. In this climate, ‘softer’ approaches may be more attractive than their ‘harder’ counterparts, since taxes and bans are frequently perceived as infringements on personal freedom. The tension between personal freedom and promoting public health has been debated since the 19th century, when government control over local water and sewage systems and the prohibition of drinking in pubs by children was derided as ‘paternalistic’ and ‘despotic’ [31]. Nowadays, government public health policies such as mandatory seat belts, scooter helmets and age limits for alcohol and tobacco use are widely accepted. The governmental impact on public health is currently highlighted by the measures taken during the COVID-19 pandemic. When danger is as obvious as in this pandemic, actions taken by authorities to protect their citizens are widely accepted—even though the disease burden of COVID-19 is clearly lower than that of CVD. Incorrectly, the urgency of CVD may be perceived as low since ‘danger’ is slow and perhaps more familiar. In addition, instead of framing government interference as a loss of individual freedom, it can also be seen as protecting individual freedom that would otherwise be manipulated by the influence of the unhealthy commodity industry [32, 33]. In fact, most lifestyle choices are made habitually and in response to environmental cues [34], prompted by actors less concerned about population (and individual) health. This is especially important in socio-economically deprived groups, where lifestyle ‘choices’ are frequently constrained by limited resources [33, 35].

In conclusion, there is convincing evidence for the effectiveness of government policies resulting in the prevention of CVD. Population-level interventions targeting the context in which lifestyle behaviour develops are especially promising, facilitating the initiation of and adherence to a healthy lifestyle. Although healthcare professionals will remain on call as firefighters, we simultaneously call upon the government to take its responsibility to prevent fires.

## Supplementary Information

Table 1. Examples of ‘soft’ and ‘hard’ policies addressing tobacco smoking, alcohol consumption, physical (in)activity and dietary behaviours, including the classification whether it is current or partial policy according to National Prevention Agreement in the Netherlands.
