# Supplementary material for: An appeal to our government for nationwide policies in the prevention of cardiovascular disease
Source: Neth Heart J. 2021 Oct 4;30(1):58–62. doi: 10.1007/s12471-021-01628-w (PMC8489361; doi:10.1007/s12471-021-01628-w)
Supplement: Supplementary file 1 — Table 1. Examples of ‘soft’ and ‘hard’ policies addressing tobacco smoking, alcohol consumption, physical (in)activity and dietary behaviours, including the classification whether it is current or partial policy according to National Prevention Agreement in the Netherlands. [file 12471_2021_1628_MOESM1_ESM.docx]

**Table 1** Examples of ‘soft’ and ‘hard’ policies addressing tobacco smoking, alcohol consumption, physical (in)activity and dietary behaviours

|  | Soft policies | Hard policies |
| --- | --- | --- |
| Tobacco smoking | - (Graphic) warning labels on tobacco packaging - Mass media campaigns^a^ | - Increased tobacco tax^a^ - Tobacco advertising ban^a^ - Ban on the visibility of tobacco products in stores^a^ - Ban on cigarette machines^a^ - Smoking ban^b^ |
| Alcohol consumption | - Warning labels on alcohol packaging - Mandatory warning (e.g. ‘drink responsibly’) in alcohol advertisements^b^ - Education about responsible alcohol consumption^a^ | - Increased alcoholic beverage tax - Age limits on alcohol sales^a^ - Alcohol advertising ban^b^ - Stricter licensing policy for alcohol retail |
| Physical (in)activity and sedentary behaviour | - Mass media campaign promoting the interruption of sedentary behaviours - Stair use prompts in office buildings - School policies on physical activity^b^ - Individual financial incentives | - Car-free city policies - Mandatory physical education at schools - Tax-reduction incentives promoting fitness club membership - Increased petrol tax |
| Dietary behaviours | - Mass media campaigns promoting healthy dietary behaviours^a^ - Food labelling^b^ - Nudging towards healthier food choices in canteens, sports clubs, schools, restaurants, supermarkets, etc. | - Sugar (sweetened beverage) tax - Age limits on energy drink sales - Unhealthy food and beverage advertising ban (e.g. on public transport, on television) - Ban on trans fats - Licensing policy for fast food retail |

^a^Current national-level policy or agreement according to National Prevention Agreement in the Netherlands

^b^Partial policy or agreement according to National Prevention Agreement in the Netherlands
